# The role of multislice computerized tomography angiography in assessing postoperative vascular complications in liver transplant patients

**DOI:** 10.3906/sag-1902-145

**Published:** 2019-08-08

**Authors:** Yaşar TÜRK, Artür SALMASLIOĞLU, Hadi SASANİ

**Affiliations:** 1 Department of Radiology, Faculty of Medicine, Namık Kemal University, Tekirdağ Turkey; 2 Department of Radiology, İstanbul School of Medicine, İstanbul University, İstanbul Turkey

**Keywords:** Vascular complication, liver, liver transplantation, CT angiography, Doppler

## Abstract

**Background/aim:**

Vascular complications can be detected in liver transplant patients. Digital subtraction angiography has served as the gold standard to make this diagnosis; however, due to its invasive nature, ultrasonography is used for the preliminary evaluation. The purpose of this study was to evaluate the role of multislice computerized tomography angiography (MSCTA) in the detection of vascular complications of symptomatic and asymptomatic liver transplant patients and to compare the results with Doppler ultrasound (Doppler US) findings.

**Materials and methods:**

Fifty-three liver transplant patients (6 symptomatic, 47 asymptomatic) underwent Doppler US examination followed by an MSCTA. The findings in each modality were interpreted in a blinded fashion and then compared.

**Results:**

MSCTA detected 15 abnormalities, none of which were detected by Doppler US. There were hepatic and splenic artery aneurysms (n = 4) and various stenoses (n = 4), infrarenal aortic anastomosis (n = 4), vena cava inferior thrombosis (n = 1), arteriovenous malformation (n = 1), and esophageal varices (n = 1).

**Conclusion:**

MSCTA detected more lesions and we believe that it should be considered as a road map for Doppler US follow-ups as well as a routine screening modality for early detection of vascular complications in symptomatic and asymptomatic liver transplantation patients that may be missed by Doppler US.

## 1. Introduction

Human whole-liver transplantation as a therapeutic option for end-stage liver disease was pioneered in 1963 by Starzl et al. [1]. Despite obstacles, liver transplantation has emerged as the best therapeutic choice for selected irreversible liver failure patients almost 50 years ago [2]. Although initial efforts were unsuccessful, today, following years of modification of surgical techniques and the introduction of new immunosuppressive agents, liver transplantation is an accepted and successful therapy for end-stage liver failure. 

Vascular complications, including hepatic artery stenosis and thrombosis, can be detected in liver transplant patients. Digital subtraction angiography (DSA) has served as the gold standard to make this diagnosis, however, due to its invasive nature, ultrasonography is used for the preliminary evaluation. 

Three-dimensional helical computed tomographic arteriography (3D CTA) with maximum intensity projection, shaded surface display techniques, and volume rendering technique (VRT) has been compared with DSA. 

The purpose of this study was to evaluate the role of multislice CT angiography (MSCTA) in the detection of vascular complications of symptomatic and asymptomatic liver transplant patients and to compare the results with Doppler ultrasound (Doppler US) findings.

## 2. Materials and methods

Fifty-three liver transplant patients were evaluated radiologically. Two radiologists (with 2 and 3 years of experience) evaluated Doppler US findings using the Logiq P5 ultrasound machine (General Electric, Boston, MA, USA) with a 3.5 MHz transducer. Of the 53 patients, 20 (37.7%) were female and 33 (62.3%) were male. The ages of the females ranged from 2 to 73 (mean: 37, standard deviation: 20) and the males between 1 and 66 (mean: 30, standard deviation: 23). The mean time between imaging and transplantation was 1082.8 days (2–4497 days, standard deviation: 938.9 days). 

After analyzing the total results regarding transplantation indications, viral etiology had the highest ratio in liver diseases, which is also the case across the country. Other indications for transplantation are shown in Table 1. 

**Table 1:** Indications for transplantation.

Indications for transplantation	Number of patients
Viral hepatitis	12
Liver metabolic disease	10
Cholestatic disease	4
Pediatric liver transplantation	3
Autoimmune hepatitis and cirrhosis	2
Hepatobiliary malignancy	2
Retransplantation	2
Alcoholic liver disease	1
Others	17
Total	53

In 34 patients (64.2%) grafts were obtained from a cadaver, and in 19 patients (35.8%) grafts from living donors were used. Of the 34 grafts taken from cadavers, 33 (97.1%) were transplanted entirely, while in 1 patient (2.9%), only the left lateral lobe lateral segment was transplanted. In 13 of the living donors (68.4%), the left lobe lateral segment was transplanted, and in 6 (31.6%) the right lobe was transplanted. Two patients (3.8%) underwent retransplantation. Four of the 53 patients (7.5%) died after transplantation due to complications. 

Computerized tomography (CT) scans were performed with a 4-detector Siemens Somatom 4 (Siemens, Erlangen) MDCT device. We obtained images of the liver with a slice thickness of 7 mm and 0 mm slice gap with noncontrast CT. The proximal descending aortic at celiac axis level was defined for use in the bolus test method in order to calculate the optimal contrast time. Iopromide 370 (ultravist) in the amount of 0.3 mL/kg was injected at a rate of 5 mL/s. 

Fifteen axial images were obtained at intervals of 1 s following a 10-s delay. The bolus test method was used to determine the required delay following contrast material injection to achieve a maximal enhancement in the arterial phase (12–25 s). This delay time, which was determined by the bolus test method, was determined as the start of the scan after infusion of 150–180 mL of Ultravist 370. This time varied between 12 and 25 s for the arterial phase. 

All reconstructions were performed by an experienced radiologist in 3D postprocessing techniques and lasted approximately 15 min. The VRT technique was used in each patient. Axial section images were manually selected only for the aorta, celiac axis, hepatic artery, left gastric artery, splenic and superior mesenteric arteries and related volumes, to include liver. Then, CT angiograms were reconstructed with the VRT technique using the lower threshold value of 70–115 HU. The radiologist who performed the VRT subjectively adjusted the appropriate window spacing, opacity, and brightness values. VRT images were obtained in projections that would best show the course of the hepatic vessels. Right anterior oblique, inferior, posterior, and lateral projections were used as standard projections in each patient. 

Reconstructed axial images and 3D VRT images were prospectively assessed by the radiologist and vascular anatomy of the liver and vascular complications (stenosis, thrombosis, pseudoaneurysm, or aneurysm) were recorded. The hepatic artery anatomy was classified according to the existing hepatic artery anastomosis. 

Fifty-three liver transplant patients (6 symptomatic, 47 asymptomatic) underwent Doppler US examination followed immediately by MSCTA using a 4-detector MSCT unit. The findings in each modality were interpreted in a blinded fashion and then compared. The study was approved by the local research ethics committee. All the patients provided written informed consent before participating in the study. 

### 2.1. Statistical analysis 

Demographic data were collected via patient medical record chart. Statistical analyses were performed using SPSS statistics software (Version 17; IBM, Armonk, NY, USA). Descriptive statistics such as mean, median, and standard deviation were used to define continuous variables. 

## 3. Results

**Figure 1:**
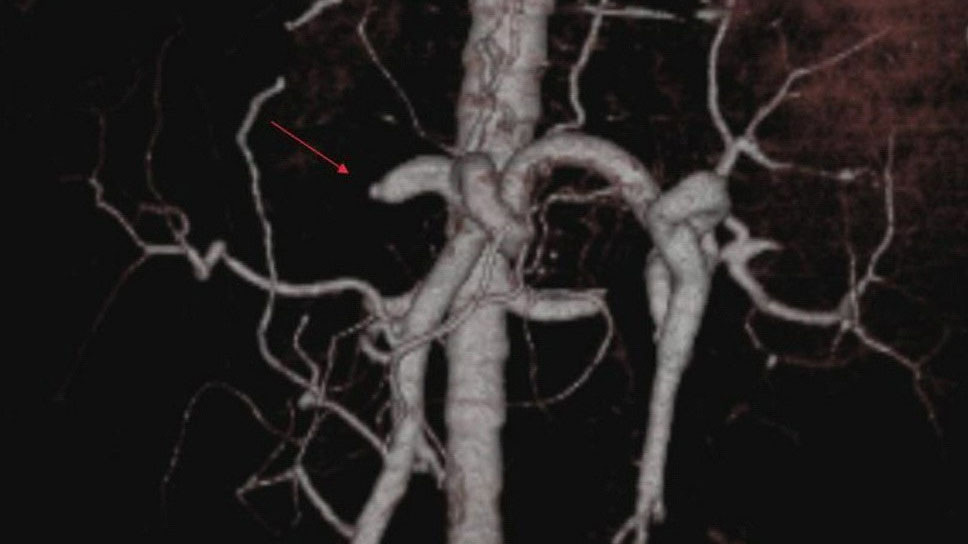
Occlusion in common hepatic artery, anastomosis between proper hepatic artery and superior mesenteric artery. VRT image demonstrates occlusion (arrow) at the common hepatic artery.

**Figure 2:**
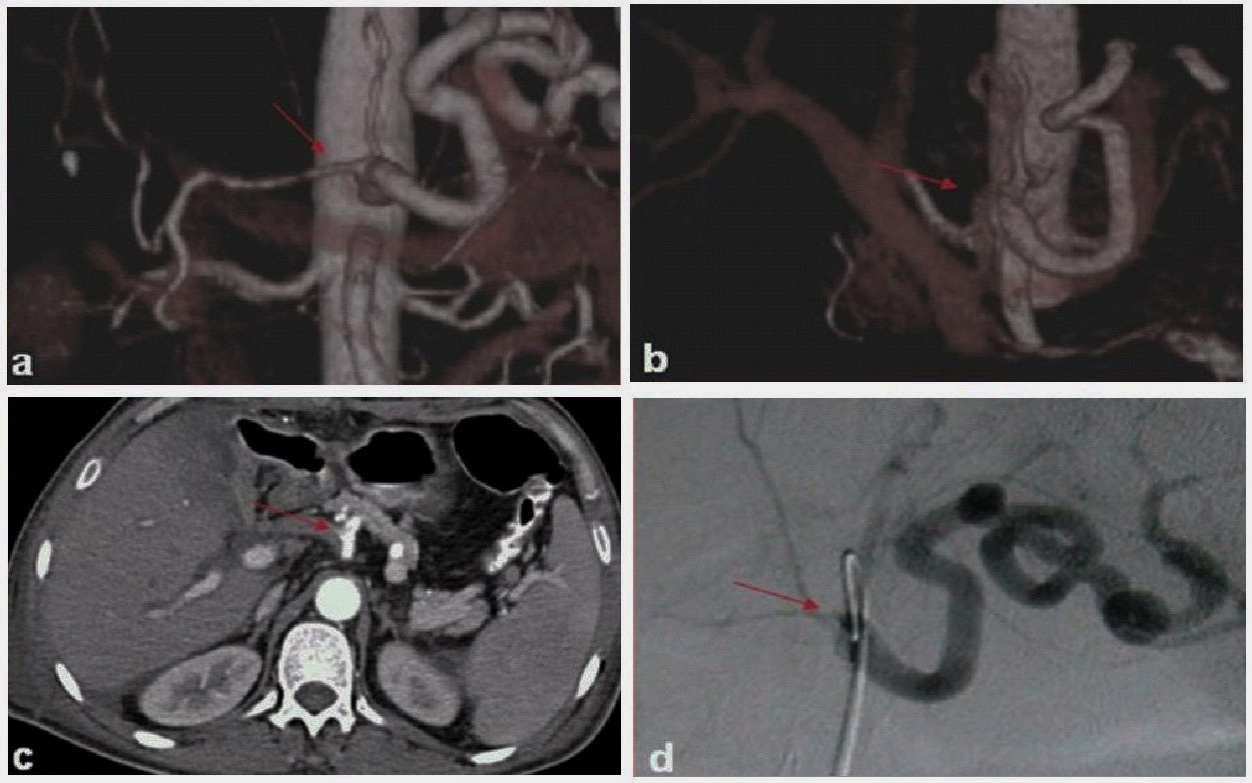
An occlusion at the common hepatic artery. (a) Preocclusion VRT image demonstrates an irregular and diffuse low-grade stenosis (arrow). Follow-up examination 2 weeks later demonstrates occlusion in (b) VRT, (c) axial CT, and (d) DSA images.

**Figure 3:**
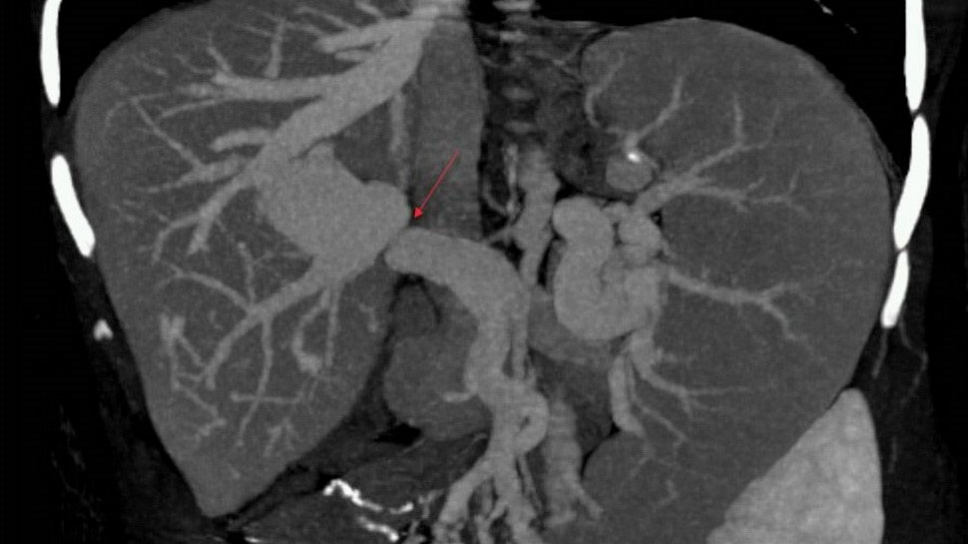
There is severe stenosis of portal venous anastomosis and marked poststenotic dilatation in an asymptomatic case. A reconstructed MIP image demonstrates severe stenosis (arrow) and significant poststenotic dilatation at portal venous anastomosis.

**Figure 4:**
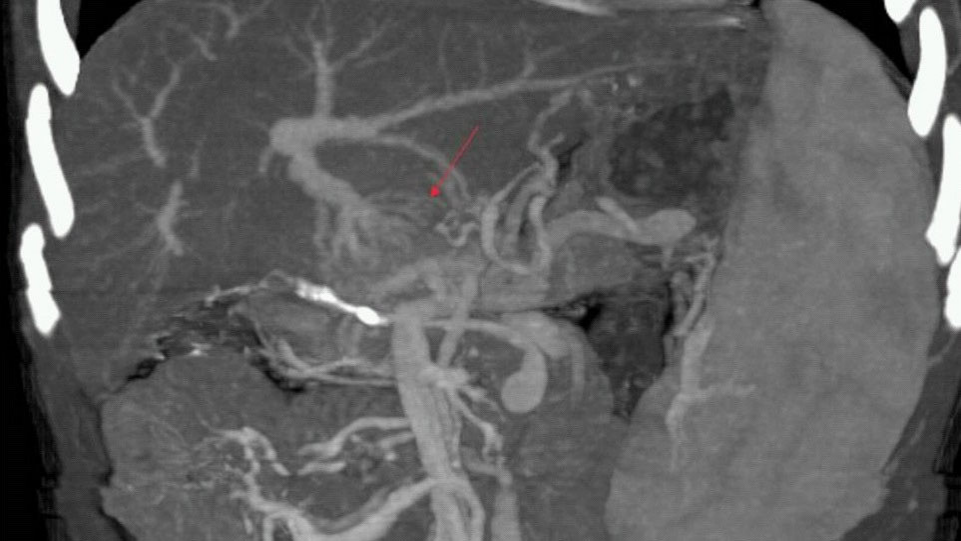
Portal vein thrombosis and cavernous transformation are seen in an asymptomatic case. A reconstructed MIP image demonstrates portal venous thrombosis and cavernous transformation (arrow).

**Figure 5:**
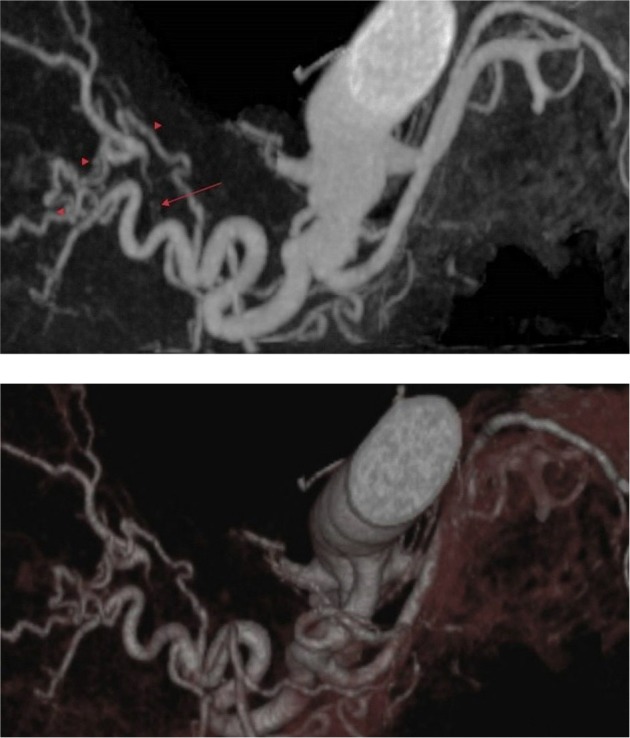
Thrombosis in the left hepatic artery. Thrombosed segments and collaterals can be seen on the MIP and VRT images of the patient who are thought to be patented by Doppler USG due to collaterals. (a) A reconstructed MIP image demonstrates thrombosis (arrow) at the proximal segment of the left hepatic artery and distal reperfusion with collateral branches (arrowheads), (b) VRT image of the same patient.

**Figure 6:**
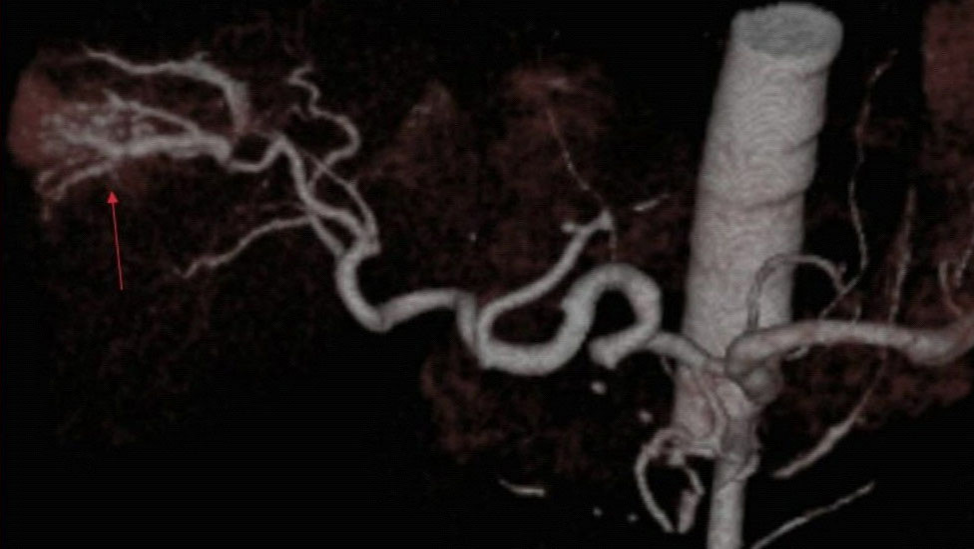
VRT findings in patients with arterioportal fistula after blind biopsy without image mapping. VRT image demonstrates an arterio-portal fistula (arrow) following a TRU-CUT biopsy.

**Figure 7:**
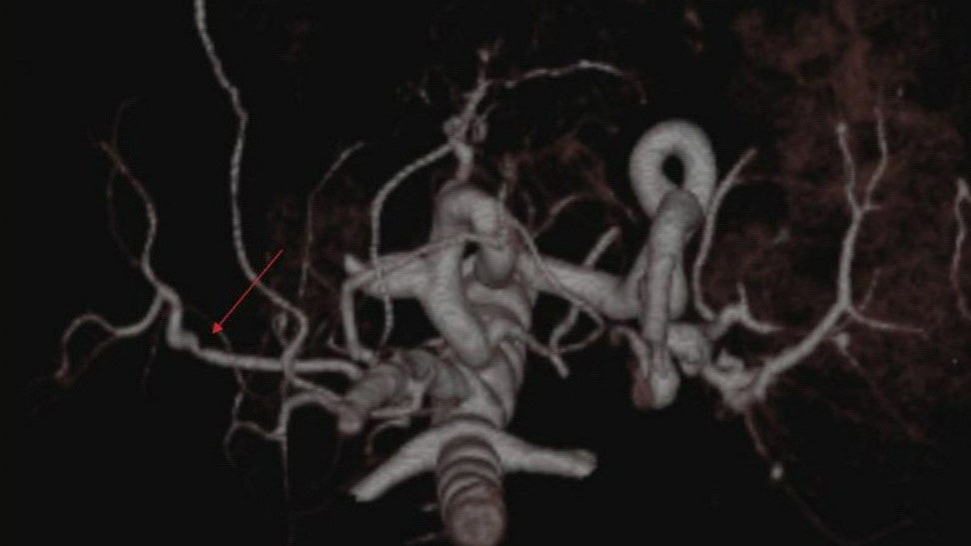
Asymptomatic stenosis of the anastomosis between proper hepatic artery and superior mesenteric artery. VRT image demonstrates stenosis (arrow) at the anastomosis between proper hepatic artery and superior mesenteric artery branch.

**Figure 8:**
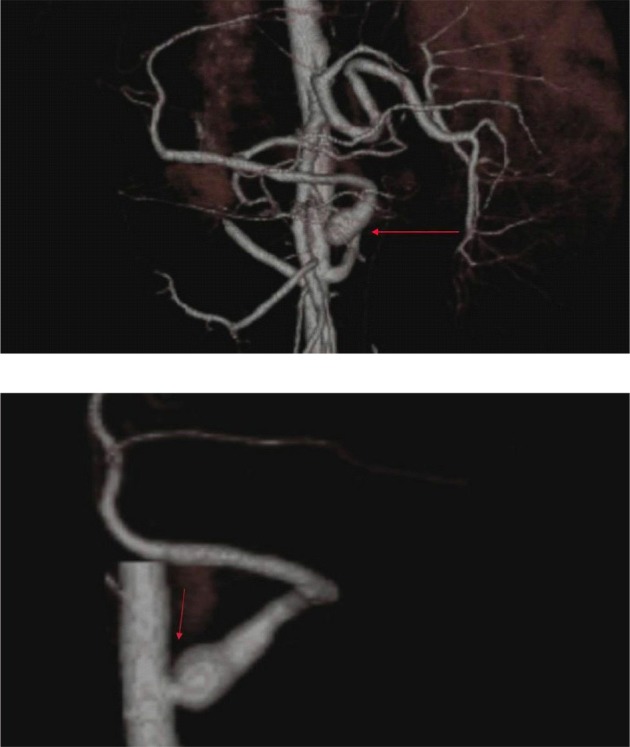
Suprarenal aortic anastomosis is followed by stenosis and poststenotic dilatation in an asymptomatic case. (a) Dilatation at the origin of the supraaortic anastomosis can be demonstrated by the VRT image, (b) VRT image in the sagittal plane demonstrates an anastomotic stricture (arrow) with poststenotic dilatation.

In 6 patients (11.3%), CTA was performed with indications (because of clinical or laboratory findings, or vascular complication suspicion in Doppler US). In the remaining 47 patients (88.7%), no clinical or laboratory findings were found to be indicative of CTA. No pathological findings were observed in 12 patients (22.6%). CTA images revealed hepatic artery aneurysm in 2 patients (3.8%), common hepatic artery thrombosis in 3 patients (5.7%) (Figures 1 and 2), infrarenal aortic anastomosis in 4 patients (7.5%), portal vein stenosis in 11 patients (20.8%) (Figure 3), portal vein thrombosis in 2 patients (3.8%) (Figure 4), stenosis in inferior vena cava at hepatic vein junction in 2 patients (3.8%), proper hepatic artery thrombosis in 4 patients (7.5%) (Figure 5), proper hepatic artery and common hepatic artery stenosis in 9 patients (17%), inferior vena cava thrombosis in 1 patient (1.9%), splenic artery aneurysm in 2 patients (3.8%), air in the intrahepatic bile ducts in 3 patients (5.7%), arteriovenous fistula in 1 patient (1.9%) (Figure 6), splenomegaly in 3 patients (1.9%), stenosis of superior mesenteric artery and proper hepatic artery anastomosis in 1 patient (1.9%) (Figure 7), hemangioma in 1 patient (1.9%), esophageal varices in 1 patient (1.9%), stenosis in suprarenal anastomosis in 1 patient (1.9%) (Figure 8), stent in the biliary tract in 1 patient (1.9%), incisional hernia in 1 patient (1.9%), intraabdominal and perihepatic free fluid in 4 patients (7.5%), azygosplenic shunt in 1 patient (1.9%), and hepatic infarction in 1 patient (1.9%). 

MSCTA detected 15 abnormalities (some patients had more than one abnormality), none of which were detected by Doppler US. They were: hepatic and splenic artery aneurysms (n = 4) and various stenoses (n = 4), infrarenal aortic anastomosis (n = 4), vena cava inferior thrombosis (n = 1), arteriovenous malformation (n = 1), and esophageal varices (n = 1). 

MSCTA also detected: thrombosis of proper hepatic artery in 4 patients (2 were detected by Doppler US), portal venous (PV) stenosis in 11 patients (3 were detected by Doppler US), PV thrombosis in 2 patients (none were detected by Doppler US and 1 was false positively detected), stenosis of proper hepatic artery and common hepatic artery in 9 patients (3 were detected by Doppler US). Twelve patients did not have any pathological findings. In 22 patients, Doppler results were in correlation with MSCTA. Correlation of Doppler US results with CTA is shown in Table 2. 

**Table 2:** True and false positivity and negativity of Doppler US results when CTA is accepted as gold standard.

Pathologies	Doppler US			
	TP	FP	TN	FN
Hepatic artery aneurysm	0	0	0	2
Infrarenal aortic anastomosis	0	0	0	1
Vena cava inferior stenosis	0	0	0	2
Vena cava inferior thrombosis	0	0	0	1
Splenic artery aneurysms	0	0	0	2
Arteriovenous malformation	0	0	0	1
Stenosis at the anastomosis between proper hepatic artery and a branch of superior mesenteric artery	0	0	0	1
Stenosis at suprarenal aortic anastomosis	0	0	0	1
Thrombosis at proximal segment of left hepatic artery and distal reperfusion with collaterals	0	0	0	1
Thrombosis of common hepatic artery	2	0	0	3
Portal venous (PV) stenosis	3	0	0	8
PV thrombosis	0	1	0	2
Stenosis of proper hepatic artery and common hepatic artery	3	0	0	5

TP, true positive; FP, false positive; TN, true negative; FN, false negative.

## 4. Discussion 

Liver transplantation is the only treatment for many patients with end-stage liver failure [3]. In line with the findings of the recently published study by Zeytunlu et al. [4], viral etiology was the leading cause of transplantation indications in our study, which is also the case across the country. Unlike patients with renal insufficiency, there is no alternative treatment such as hemodialysis or peritoneal dialysis. Nowadays when the number of donors is very limited, selection of suitable candidates for transplantation is very important. This choice is made with a multidisciplinary approach and strict rules. Another important factor is that the complications that may develop in the posttransplant stage can be caught without irreversible clinical and histopathological symptoms, and necessary interventions can be made. Vascular thrombosis or stenosis, biliary obstruction, bleeding, posttransplant stage rejection, and posttransplant neoplasia are serious complications [5]. Most complications lead to morbidity and mortality in the posttransplant stage. However, some complications may not cause symptoms. Such complications can lead to serious problems for the patient in advanced stages if they are overlooked. For this reason, it is of great importance to detect all forms of complications in an objective way. 

The role of radiology in this phase is very important. Ultrasonography is the most commonly used method for evaluating transplant patients in the early postoperative period. Ultrasonography is a valuable method because it does not require the patient to be mobilized and allows the liver parenchyma and bile ducts to be evaluated. Doppler US provides an assessment of vascular structures, but false negative results can be encountered [6,7]. For instance, in less than 72 h after transplantation, increased hepatic artery resistance (resistive index >0.8) due to a prolonged period of ischemia or older donor age can cause misinterpretation [8]. Furthermore, one of the most important problems associated with Doppler US is its operator dependability. In the early postoperative period, patients must be assessed under intensive care unit conditions. Ambient conditions are usually suboptimal for ultrasonography. In addition, multiple large incisions and drains can be found in the anterior wall of the patient’s abdomen. Incisions make probe manipulation difficult and complicate compliance with standard examination plans. This can lead to poor visualization of all liver segments and vascular structures. Cooperation with patients in the pediatric age group may not be possible. Doppler sensitivity of the user device may not be sufficient. The presence or absence of a flow in a vessel is not enough for an effective Doppler US report. Reduced blood flow due to low cardiac output or vasospasm may cause flow loss in the Doppler US. Flow velocities and patterns should be clearly demonstrated, and index measurements should be made. However, these measurements may lead to misleading results if not in accordance with the rules [9,10]. Ultrasound with microbubble contrast may increase the visibility of the patent low flow hepatic artery [11]. Doppler studies have shown that significant variability among operators can be found. In order to remove this disparity, it may be necessary for all patient studies to be done by the same person, or for all operators to be trained with certain coordination. It is more difficult for some pathologies to be recognized by ultrasonography or Doppler. For example, a hematoma in an early stage may not be recognized ultrasonographically. The presence of hepatic arterial thrombosis, a very important complication, cannot be established if collateral vascular structures develop [12]. If such conditions lead to clinical symptoms, further imaging modalities are sought to reveal the anomaly. Digital subtraction angiography (DSA) is used as an advanced modality in the conventional approach. Although DSA is accepted as a gold standard, it is an invasive method and should be used only for revealing vascular pathologies that cannot be explained by other modalities, or for the confirmation of an anomaly identified by Doppler. Stell et al. [13] performed Doppler ultrasonography in 51 patients who underwent liver transplantation and reported many anomalies in the organ vessels during the short follow-up (posttransplant, twice in the first week) that did not lead to any clinically significant results. DSA for the purpose of verifying the anomalies of this type or for clarification can be regarded as an unnecessary attempt to harm the patient. The principle of preferring a noninvasive modality to invasive modalities in cases where a therapeutic intervention cannot be performed is becoming increasingly accepted today, and it is at the forefront in evaluating alternative modalities to DSA in transplanted patients. 

One of the modalities that can be used as an alternative to DSA is magnetic resonance angiography (MRA). In many studies comparing MRA with DSA, it has been reported that the results correlate with each other [14,15]. Nevertheless, there are few studies in transplant patients [16,17]. One of the important reasons for this is that patient-connected devices (especially monitors) in early-stage intensive care conditions are not compatible with MRA. Studies generally indicate that MRA is a sensitive method but the specificity is low. 

Computed tomography is another modality that can be used at a posttransplant stage. Initial studies on the use of this modality in patients with liver transplantation have begun towards the end of the 1980s. Letourneau et al. [18] reported that CT is a valuable modality in evaluating graft integrity and investigating extrahepatic fluid presence. Similar studies have been reported by Schurawitzki et al. [19], Marincek et al. [20], and Shyn et al. [21], and the same results were obtained. A common conclusion of these studies is that CT should not play a crucial role in assessing vascular structures, and when vascular problems are considered, DSA and Doppler should be used. The fact that the spatial and temporal resolutions of the tomography devices used in this period were insufficient and the computer technology was not advanced enough could be considered as the main reason. As technology advanced, studies have begun to show that CT can also be used for vascular evaluation. By using the maximum intensity projection (MIP) technique, Legmann et al. [22] reported that hepatic arterial thrombosis could be demonstrated with high sensitivity (100%) and specificity (92%) in transplant patients. In this study, the sensitivity of Doppler was found to be 100%. The thromboses observed in the study (verified with DSA) may have similar sensitivity with both modalities because they either affected the entire distal segment starting from an anastomosis or had extensive thrombosis involving all hepatic arteries. 

Hidajat et al. [23] compared DSA with BTA, MIP, and shaded surface display (SSD) in the preoperative evaluation of transplant candidates. In this study, it was reported that CTA was able to provide as much information as DSA, and even more, and SSD was quite successful in showing the vascular structures. 

Revolutionary advances in CT imaging have begun with the introduction of multidetector computerized tomography (MDCT). It became possible to scan much wider areas in much shorter times and with thinner slice thicknesses. Thanks to these possibilities, arterial, portal, and venous phases can be visualized more precisely. This facilitated vascular evaluation. Furthermore, the ability to take thinner sections allowed the three-dimensional reconstructions to be more visually successful and more frequent for diagnostic purposes. Kamel et al. [24] reported that MDCT assessment of liver transplant candidates had a successful evaluation of both parenchyma and vascular structures, and volumetric measurements could be made at the same time. Developing computer technology has also contributed to this improvement, and three-dimensional processes that require high processing power have become real-time or near real-time. An advantage of the VRT technique is that it allows visualization without causing any data loss. In the SSD technique, in which three-dimensional images are also obtained, the voxel between certain threshold HU values is visualized, while others become completely transparent. This technique does not allow multiple tissue types to be displayed at the same time, or features such as semitransparency. VRT is independent of these constraints and provides the closest views to the actual anatomy. Initial studies on the use of VRT in liver transplant patients have been reported by Katyal et al. [25]; frequent and sometimes fatal complications have been detected by this technique. Following a preliminary study by Brancatelli et al. [26], the sensitivity of VRT to vascular lesions was calculated to be 100%, specificity 89.8%, PPD 92%, and NPD 100%. A study that partially contradicts the results of these studies was done by Byun et al. [27]. In this study, MIP and VRT methods were compared with each other in the evaluation of hepatic arterial anatomy in MDCT performed in potential liver donors, and it was concluded that MIP was more successful than VRT in assessing anatomical variations and took less time. However, in this study the structures outside the hepatic artery were not evaluated and the additional contributions of the two methods to the clinician were not discussed. Piccoli et al. [28] pointed out that using both MIP and VRT technique images could replace conventional angiography in their study. According to Michels’ classification, in type I the hepatic artery supplies the right and left lobs through the right and left hepatic arteries. However, in variative conditions, arterial blood flow could originate from superior mesenteric, left gastric, or directly from the aorta, either alone or in combination with Michels’ type I [29]. Considering the application of different operational techniques due to the variation of the vasculature, we can predict how difficult it is to evaluate these cases by ultrasound alone. As can be seen from the studies, MDCT and postprocessing procedures are increasingly playing a role in the pre- and postoperative follow-up of transplant patients and competing with DSA. In addition to evaluating the raw images, the use of three-dimensional visualization techniques such as MIP and VRT has increased diagnostic accuracy. It is also possible to create movies in order to demonstrate 3D anatomy, which can be useful in the diagnosis and is also a better way to display the pathologies to the surgeon and the clinician. Their use in combination with each other in a particular routine gives the most successful results. In our study, we acted with the same logic and saw many symptomatic and asymptomatic pathologies that could not be detected with Doppler US. Early diagnosis of these pathologies is needed because they can lead to untreatable outcomes. For this purpose, we think that MDCT can be used as a road map. It is conceivable that Doppler follow-ups can be made more objective and operator-independent by making use of the tips provided by these images. 

In many transplantation units, including ours, radiological screening methods are not used postoperatively unless there is a clinical or biochemical anomaly in the patients. When a problem is encountered, ultrasonography and Doppler ultrasonography are used first, followed by more advanced modalities. This delay in radiological admission may cause delays in the initiation of treatment and can cause irreversible changes in the graft parenchyma and its vessels, creating serious problems that can lead to graft loss. In our study, both radiologists who performed Doppler ultrasonography on the transplant patients had many unsuccessful results in this regard, although they were the most experienced people in our unit. 

Our study has an important feature compared to other studies in transplant patients. To our knowledge, neither Doppler nor CTA has been performed at the same time in asymptomatic transplant patients in any study. Despite the absence of clinical and biochemical indications, the majority of patients had several pathologic findings, some of which were serious and some of which were mild. Most of these pathologies could only be detected with CTA. Based on this data, we think that CTA is necessary as soon as possible after transplantation with the aim of clearly drawing out a road map plane of each transplant patient and documenting the changes that occur during surgery. Routine Doppler US based on this map will be much more effective. 

This study has several limitations. First, the examination times are very variable (postoperative 1–4500 days). Second, although CT scan provides the most useful details about the underlying pathology, it may not have an obvious clinical symptom (for example, minor portal vein stenosis). Third, radiation exposure of the patient is another limitation. However, new generation CT devices with ultra-low-dose radiation lead to less than expected exposure of the patient. Larger studies with long-term follow-up are required in order to demonstrate the statistical significance of the possible contribution of an earlier diagnosis provided by CTA. 

Liver transplantation is an accepted and successful form of treatment for a variety of irreversible acute and chronic liver diseases. Comprehensive patient care, advances in surgical technique, and the development of new immunologic agents have all led to a decrease in mortality and morbidity from liver transplantation. Radiologists play a vital role in the postoperative care of transplant recipients. CTA is a safe, noninvasive, accurate, and reliable method that can be used to show patency, stenosis, or thrombosis of the hepatic artery in liver transplant patients and to assess the presence and extent of damage to liver parenchyma. In our study, MSCTA detected more lesions compared to Doppler US and we believe that it should be considered as a road map for Doppler US follow-ups and as a routine screening modality for early detection of vascular complications in symptomatic and asymptomatic liver transplant patients that may be missed by Doppler US. Recent technological advancements permit the construction of MSCT units with more detectors, which will increase MSCTA’s sensitivity, specificity, and its ability to detect smaller pathologies at earlier stages as well as contrast dose and radiation dose reduction.
